# Reducing perinatal mortality among Indigenous babies in Queensland: should the first priority be better primary health care or better access to hospital care during confinement?

**DOI:** 10.1186/1743-8462-2-11

**Published:** 2005-05-27

**Authors:** Trisha Johnston, Michael Coory

**Affiliations:** 1 Epidemiology Services Unit, Health Information Branch, Queensland Health GPO Box 48 Brisbane Queensland 4001 Australia

## Abstract

**Background:**

The perinatal mortality rate among Indigenous Australians is still double that of the rest of the community. The aim of our study was to estimate the extent to which increased risk of low birthweight and preterm birth among Indigenous babies in Queensland account for their continuing mortality excess. If a large proportion of excess deaths can be explained by the unfavourable birthweight and gestational age distribution of Indigenous babies, then that would suggest that priority should be given to implementing primary health care interventions to reduce the risk of low birthweight and preterm birth (eg, interventions to reduce maternal smoking or genitourinary infections). Conversely, if only a small proportion is explained by birthweight and gestational age, then other strategies might need to be considered such as improving access to high-quality hospital care around the time of confinement.

**Methodology:**

Population-based, descriptive study of perinatal mortality rates among Indigenous and non-Indigenous babies, in Queensland, stratified by birthweight and gestational age.

**Results:**

Indigenous babies are twice as likely to die as their non-Indigenous counterparts (rate ratio1998–2002: 2.01; 95%ci 1.77, 2.28). However, within separate strata of birth weight and gestational age, Indigenous and non-Indigenous rates are similar. The Mantel-Haenszel rate ratio adjusted for birth weight and gestational age was 1.13 (0.99, 1.28). This means that most of the excess mortality in Indigenous babies is largely due to their unfavourable birth weight and gestational-age distributions. If Indigenous babies had the same birth weight and gestational age distribution as their non-Indigenous counterparts, then the relative disparity would be reduced by 87% and 20 fewer Indigenous babies would die in Queensland each year.

**Conclusion:**

Our results suggest that Indigenous mothers at high risk of poor outcome (for example those Indigenous mothers in preterm labour) have good access to high quality medical care around the time of confinement. The main reason Indigenous babies have a high risk of death is because they are born too early and too small. Thus, to reduce the relative excess of deaths among Indigenous babies, priority should be given to primary health care initiatives aimed at reducing the prevalence of low birth weight and preterm birth.

## Background

From a public-health perspective, the major obstetric and perinatal problem in Australia is the poor health of Indigenous mothers and babies. Although Australia as a whole has one of the lowest perinatal mortality rates (PMR) of any of the established market economies [1], Indigenous Australians have PMRs that are at least twice as high as their non-Indigenous counterparts [2,3].

Moreover, the relative excess of deaths among Indigenous babies has not improved over time. In Queensland in 1987–1989, the PMR was 2.2 times higher among Indigenous compared with non-Indigenous babies and in 2000–2002 the rate was still 2.1 times higher. A similar lack of progress is evident in other states [4-6].

The risk of perinatal death is strongly related to a baby's birthweight and gestational age. For example, the risk of death for a moderately preterm baby (33 to 36 weeks gestation) in Queensland is 8.2 times greater than that of a term baby and for a very preterm baby (27 to 32 weeks) the risk is 41.3 times greater. Similarly, a baby who was born at term but weighed less than 2500 g was 6.3 times more likely to die than a baby who weighed more than 2500 g. In Queensland, Indigenous babies are 1.6 (95%ci: 1.5,1.7) times more likely to be born preterm (<37 weeks gestation) and 2.2 (2.0,2.5) times more likely to be low birthweight at term than non-Indigenous babies [3]. Similar disparities in birthweight and gestational age have been reported from the other states of Australia [2,5,7-10].

The aim of this paper is to estimate the number and proportion of excess deaths among Indigenous babies that can be explained by their higher risk of low birthweight and preterm birth. This analysis strategy assumes that the distribution of birthweight and gestational age among Indigenous babies reflects the prevalence of antenatal risk factors such as smoking, infection, maternal nutrition and psycho-social stress; while any excess of mortality that remains after the excess low-birthweight and preterm risk among Indigenous babies is removed might say something about the quality of medical care at the time of confinement.

Although such reasoning has many proponents [11-13], risk factors such as smoking and infection are likely to also have at least a small effect on mortality that is independent of birthweight and gestational age. That is, adjustment of perinatal mortality rates by birthweight and gestational age is not a perfect way of assessing the quality of medical care at the time of confinement. It is similar to case-mix adjustments used in other settings to allow for differences in risk [14,15]. Such adjustments are not expected to remove all confounding. Instead, the reasoning is that the adjusted rates, although not perfect, provide a useful way of identifying policy issues, setting agendas, and facilitating discussion.

To be more specific, if a large proportion of excess deaths can be explained by the unfavourable birthweight and gestational age distribution of Indigenous babies, then that would suggest that priority should be given to implementing primary health care interventions to reduce the risk of low birthweight and preterm birth (eg, interventions to reduce maternal smoking or genitourinary infections). Conversely, if only a small proportion is explained by birthweight and gestational age, then other strategies might need to be considered such as improving access to high-quality hospital care around the time of confinement.

## Methods

Data were obtained from the population-based Queensland Perinatal Data Collection for the five years 1998 to 2002. This was the most recent five-year period for which complete data were available. The database includes information on all livebirths and stillbirths of at least 20 weeks gestation or 400 g birthweight. A perinatal death is defined as a stillbirth or the death of a liveborn baby within 28 days of birth. Our data set comprised 231,039 births and 2,255 deaths to non-Indigenous mothers and 13,920 births and 273 deaths to Indigenous mothers. Indigenous status is based on the self-reported Indigenous status of the mother and the gestational age is based on the best clinical estimate, which might be derived from the date of the last menstrual period, ultrasound in early pregnancy or maturity scoring of the neonate at birth. The method used is not recorded.

In this paper we report the results of a Mantel-Haenszel procedure, which was used to determine the relationship between perinatal mortality and Indigenous status adjusted for the effect of low birthweight and preterm birth. The proportion and number of deaths that could be avoided were estimated by comparison of the crude and adjusted rates.

We also used Poisson-regression models to adjust perinatal mortality rates for birthweight and gestational age. We variously fitted single week or four week categories of gestational age and 250 g and 500 g categories of birth weight. The results were the same as using the Mantel-Haenzel approach with broad categories of gestational age and birthweight, and we included these in preference to the Poisson-regression models for ease of interpretation.

## Results

**Figure 1:**
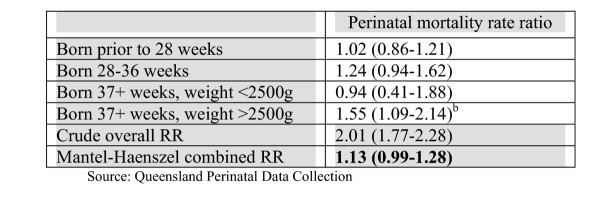
Rate ratios^a ^comparing Indigenous to non-Indigenous perinatal mortality stratified by preterm and birthweight status, 1998–2002. a) Rate ratios greater than 1.0 indicate higher mortality among Indigenous babies. b) Differences between stratum-specific rate ratios are not statistically significant (χ^2 ^(3) = 5.78, p = 0.1226).

The crude perinatal mortality rate among Indigenous babies was 19.6 per 1000 births, which was 2.01 (95%ci:1.77,2.28) times higher than the rate among non-Indigenous babies. When perinatal mortality rates were compared within each birthweight and gestational age strata, the point estimates suggested that Indigenous babies were only slightly more likely to die than non-Indigenous babies (Figure 1). The test for homogeneity of the rate ratios across strata was not significant (χ^2 ^(3) = 5.78, p = 0.1226) suggesting that the effect of Indigenous status is the same across the birthweight and gestational age strata (except for statistical noise) and that it is appropriate to use the adjusted combined estimate: M-H Adjusted RR = 1.13, 0.99–1.28.

These results suggest that if the population of Indigenous babies had the same birthweight and gestational age distribution as non-Indigenous babies, then the relative disparity would be reduced by 87% and there would be 20 fewer deaths of Indigenous babies per year.

## Discussion

### Interpretation of results

Perinatal mortality among Indigenous babies has remained twice that of their non-Indigenous counterparts for more than a decade. We found that most of this mortality excess is because Indigenous babies are at greater risk of being born too early and too small. In contrast, the case fatality rates of Indigenous babies who were born preterm or of low birth weight were similar to their non-Indigenous counterparts.

Using a framework advocated by several perinatal epidemiologists [11-13], these results suggests that, broadly speaking, access to high quality care during confinement is adequate for Indigenous mothers and babies. That is, priority should initially be given to primary health care interventions to reduce the proportion of preterm and low birth weight babies.

### Policy implications

Risk factors for preterm birth and low birth weight include smoking, gentio-urinary tract infections, poor maternal nutrition and psycho-social stress [16-19]. Several studies have reported a higher prevalence of these risk factors among Indigenous compared with non-Indigenous mothers.

More specifically, the prevalence of smoking among Indigenous women during pregnancy has been reported to be more than 60%, which is at least 3 times the prevalence for non-Indigenous women [9,20,21]. A recent Cochran review found that there are effective primary health care interventions to help and support women to stop smoking that lead to fewer preterm babies and better birthweights [22]. Further, we know that Indigenous women are more than two times as likely to have a urinary tract infection during pregnancy as non-Indigenous women [23]. In overseas studies, primary health care interventions to detect and treat asymptomatic bacteruria have been shown to decrease preterm birth by 40% [24].

In Australia, the best example we have of a primary health care initiative aimed at reducing risk factors among Indigenous mothers is the Strong Women Strong Babies Strong Culture program in the Northern Territory [25]. This program resulted in increased early attendance for antenatal care, reduced numbers of STDs and a reduced proportion of low birthweight babies [26].

Although such results are encouraging, if substantial progress is to be made across the whole of Australia, a properly funded national initiative is needed. Such an initiative would include funding to improve access to culturally appropriate primary health care during the antenatal period, which would deliver, inter alia, interventions for smoking cessation, screening and treatment of genito-urinary tract infections, screening for domestic violence, and programs aimed at reducing alcohol consumption and poor nutrition.

It would not be a case of one strategy fits all. Instead local partnerships with possibly different types of service models would be needed to implement the national initiative. This approach will encourage creativity, innovation and risk taking, which will be essential ingredients to tackling a situation that has proved difficult to improve.

### Study limitations

Using vital statistics to set agendas has a long and continuing tradition in public health [27]. The advantages of such statistics are convenience, low cost and total enumeration. The disadvantages are insufficient and inaccurate data, which create uncertainty about the validity of the results [27]. This study used four variables: perinatal death, birthweight, gestational age, and Indigenous status. It is unlikely that an important number of perinatal deaths were missed because they are checked against notifications to the Registrar-General of Births, Deaths and Marriages. It is also unlikely that there are important errors in the measurement of birthweight. Consequently, the main areas of uncertainty are Indigenous status and gestational age.

With regard to Indigenous status, some mothers may be reluctant to identify as Indigenous, others may be non-Indigenous with an Indigenous male partner, or midwives may not ask the mother or make an educated guess [28]. However, of all the types of mortality data, perinatal mortality provides the most accurate estimate of excess Indigenous mortality because the numerator (number of perinatal deaths) and denominator (number of births) for the rate can be obtained from the one data set. This is in contrast to adult death rates where identification of Indigenous people can be different in death registration data (the numerator for mortality rates) and population data (the denominator). This problem of the numerator not being appropriate for the denominator is not unique to comparisons of Indigenous and non-Indigenous Australians; it hinders interpretation of race-specific rates around the world [29,30]. Thus, of all the routinely available mortality data, perinatal data provides the most valid estimate of the mortality excess for Indigenous people and provides robust support for policy discussions.

For several reasons, gestational age is known to be less accurate among Indigenous than non-Indigenous babies [31]. Nevertheless, previous work in Queensland and elsewhere has shown that gestational age in combination with birthweight provides a better statistical adjustment of mortality rates than birthweight alone [32,33]. We therefore considered it better to present birthweight and gestational age adjusted rates, rather than just birthweight-adjusted rates.

## Conclusion

Although perinatal mortality rates in Queensland have decreased over the last 16 years, the rates in Indigenous populations remain at least double those in the non-Indigenous population. Our analyses, stratified by birthweight and gestational age, suggest that the priority for reducing the excess mortality among Indigenous babies is primary health care to reduce the prevalence of risk factors during the antenatal period. A primary health care approach encompasses a much-needed component of an overall shift towards empowerment of Indigenous women and increased awareness and ownership of health which has the potential to play an important role in reducing the social inequality that has resulted in outcomes such as those found for perinatal mortality.

## Authors' contributions

TJ performed the statistical analysis and participated in drafting the manuscript. MC conceived of the study and its design and participated in drafting the manuscript. Both authors read and approved the final manuscript.
